# Seasonal differences in climate change explain a lack of multi-decadal shifts in population characteristics of a pond breeding salamander

**DOI:** 10.1371/journal.pone.0222097

**Published:** 2019-09-06

**Authors:** Mark A. Kirk, Mark L. Galatowitsch, Scott A. Wissinger

**Affiliations:** 1 Department of Zoology and Physiology, University of Wyoming, Laramie, Wyoming, United States of America; 2 Biology and Environmental Science Departments, Allegheny College, Meadville, Pennsylvania, United States of America; 3 Department of Biology, Centre College, Danville, Kentucky, United States of America; Universitat Zurich, SWITZERLAND

## Abstract

There is considerable variation among studies that evaluate how amphibian populations respond to global climate change. We used 23 years of annual survey data to test whether changes in climate have caused predictable shifts in the phenology and population characteristics of adult spotted salamanders (*Ambystoma maculatum*) during spring breeding migrations. Although we observed year-to-year correlation between seasonal climate variables and salamander population characteristics, there have not been long-term, directional shifts in phenological or population characteristics. Warm winters consistently resulted in early migration dates, but across the 23-year study, there was no overall shift towards warmer winters and thus no advanced migration timing. Warm summers and low variability in summer temperatures were correlated with large salamander body sizes, yet an overall shift towards increasing body sizes was not observed despite rising summer temperatures during the study. This was likely due to the absence of long-term changes of within-year variation in summer temperatures, which was a stronger determinant of body size than summer temperature alone. Climate-induced shifts in population characteristics were thus not observed for this species as long-term changes in important seasonal climate variables were not observed during the 23-years of the study. Different amphibian populations will likely be more resilient to climate change impacts than others, and the probability of amphibians exhibiting long-term population changes will depend on how seasonal climate change interacts with a species’ life history, phenology, and geographic location. Linking a wide range of seasonal climatic conditions to species or population characteristics should thus improve our ability for explaining idiosyncratic responses of species to climate change.

## Introduction

Despite a general consensus that many amphibian populations are declining and experiencing long-term demographic changes, there is less agreement on which stressors are the most important causal factors [1, 2, 3, 4]. The most common stressors that have been hypothesized include some combination of non-native species introductions, habitat loss, emerging diseases (e.g., chytrid fungus; [5]), and global climate change (see [6] for a review of hypotheses). The impacts from climate change on amphibians are particularly diverse and include shifts in body size [7, 8, 9, 10, 11], increased vulnerability to diseases [12], range contractions [13, 14], and population declines/extirpations [15, 16, 17, 18]. About half of amphibian studies that focus on climate change impacts have found declines in the abundance of species [19].

Climate change impacts are likely to be profound for amphibians because they are ectothermic with life cycles and life history attributes that are tightly linked to climate [20]. Temperature regulates metabolic processes for amphibians, and increasing temperatures should result in higher energetic expenditures, decreased growth rates, and lower resource allocation during overwintering periods [8, 9, 11, 21]. For amphibians that inhabit shallow, non-permanent wetlands (e.g., ponds and marshes), the hydroperiod of these habitats is tightly linked to patterns in snowmelt, seasonal rainfall, or evapotranspiration rates, all of which are predicted to change survival, recruitment, and population size [18, 22, 23]. Temperature and precipitation also serve as important triggers for the initiation of annual breeding migrations [24, 25, 26], and shifts in the timing of reproduction have been correlated with warming temperatures [27, 28].

Despite the sensitivity of amphibians to changes in climate, it has been difficult to make generalizations about the consequences of climate change because of species- and population-level differences in physiology, life history, and habitat use [27, 29]. Idiosyncratic responses to climate change are observed across many taxonomic groups [30], which make it difficult to predict amphibian responses to climate change and establish effective conservation strategies [31, 32]. Furthermore, there are considerable differences in how climate variables (temperature, precipitation, seasonal extremes) are changing at the local scale across different geographical regions as compared to trends at the global scale [33, 34]. Given the large inter-annual fluctuations that are observed in many amphibian populations, detecting population trends and their associated climatic drivers can be difficult without long-term data [1, 35, 36].

The first objective of this study was to determine whether the population characteristics and migration timing of adult spotted salamanders (*Ambystoma maculatum*), which migrate annually from terrestrial burrows to shallow, lentic habitats [24, 25, 37], responded to annual climate variation over a 23-year study. We specifically evaluated whether 1) the number of returning adults, 2) male to female sex ratios, 3) body size and 4) migration timing have responded to annual variation in climate during the seasonally active (spring migration and breeding; summer growth and larval metamorphosis) versus non-active (winter) times of the year. The second objective was to determine whether any of these attributes exhibited long-term population-level shifts, given the global patterns of rising temperatures and precipitation change associated with climate change.

We made four explicit predictions regarding the four attributes of migrating spotted salamander populations based on previously documented effects of climate on amphibians. First, we predicted that warm temperatures and low precipitation would result in small numbers of returning adults, given that higher thermal stress, desiccation, and hydrologic alterations have all been correlated with amphibian population declines [15, 16, 17, 22]. Second, we predicted that warm temperatures and low precipitation would lead to skewed sex ratios with high abundances of males because females have lower probabilities of migrating when migration period conditions are suboptimal [23, 38]. Third, we predicted that warm temperatures and low precipitation would lead to small salamander body sizes, due to the potential for increased metabolic rates [7, 9, 11]. Fourth, we predicted that warm temperatures would lead to early migration dates, given the prominent phenological shifts towards advanced migration timing that have been attributed to climate warming [27, 28, 39, 40].

## Materials and methods

### Study area and salamander collections

Spotted salamander populations were monitored annually during a 23-year period from 1995–2017 at the Bousson Environmental Research Reserve (BERR) in northwestern Pennsylvania. The reserve is a mixed deciduous-conifer old growth forest that has seen minimal to no habitat loss during the past century. Spotted salamanders were annually monitored at five ponds (41.598°, -80.042°; S1 Fig) in the BERR, which were constructed in 1988 to replace a failing impoundment that had created a small shallow lake. In addition to spotted salamanders, wood frogs (*Lithobates sylvaticus*), leopard frogs (*L*. *pipiens*), green frogs (*L*. *clamitans)*, American toads (*Anaxyrus americanus*), and red-spotted newts (*Notophthalmus viridescens*) are observed occupying the ponds [41]. The five ponds are similar in size (70–152 m^2^ surface area), depth (1 m maximum depth), hydroperiod (semi-permanent: only drying during the driest of years and do not sustain fish populations), and are located within 100 m of each other in the riparian zone of a 3^rd^ order stream (S1 Fig).

Adult spotted salamanders were captured during annual migrations from February to May using aluminum drift fences and pitfall traps. Separate fences surrounded ponds P1and P2, P3 and P4, and P5 (S1 Fig). The aluminum fences were buried ~15 cm in the ground and extended 30–40 cm above the ground. Pitfall traps consisted of 4-liter buckets that were buried flush to the ground and positioned along the fences every 3–5 meters. Although the migration period varied from year to year, pitfall traps were opened on evenings with forecasted precipitation in late winter–early spring after the ground had largely thawed and ice had melted off the ponds, as these are important stimulants for spotted salamander movement [24, 36]. We checked the traps each morning at daybreak (5:00–7:00) and recorded the trap location (pond), date, sex, body weight (g), total length (mm), and snout-vent length (SVL; mm) for all individuals. All salamanders were then released into the pond corresponding to their trap location. Handling and processing of salamanders was approved by the Institutional Animal Care and Use Committee (IACUC) at Allegheny College.

### Climate data

We acquired daily climate data for these breeding ponds for all years of our study using the Daily Surface Weather and Climatological summaries database (Daymet; [42]). Daymet provides daily estimates of six climatic variables for 1 km by 1 km gridded cells based on latitude and longitude. The six climate variables and their respective units are precipitation (mm/day), solar radiation (watts/m^2^), snowpack coverage (kg/m^2^), maximum daily temperature (°C), minimum daily temperature (°C), and water vapor pressure (i.e., humidity; pascals). Daymet climate data have been previously used for evaluating the effects of climate on fish and amphibian populations [18, 43].

We sorted climate data into three ‘climatic periods’ of interest: climate conditions during the active summer season (hottest period), the non-active winter season (coldest period), and during the active spring migration. Summer (SU) was defined as the period from June 21-September 20 and winter (WI) was defined as the period from December 21-March 20. We focus on summer and winter conditions in particular because we expect these seasons to be the most thermally stressful [31, 32]. The springtime migration period (SM) was defined as the period in which 95% of all salamanders were captured. Using the 95^th^ percentiles eliminated the potential of outliers with very early migration dates from having an influence on results [27, 28]. 95^th^ percentiles were highly correlated with first observed migration dates for males and females (both *r* ≥ 0.88) and 95^th^ percentiles had higher performance when predicting migration timing compared with first migration date. We only focus on the Spring migration period rather than the entire Spring season because 1) of the importance of local climate conditions during the actual migration period (e.g., precipitation; [24, 37]) and 2) migrations typically span the Winter to Spring transition period (early March to end of April), and thus springtime season conditions would be more reflective of post-migration conditions.

We calculated the mean and the coefficient of variation (CV) for both minimum daily temperature and daily precipitation in the three ‘climatic periods’ to be used as predictor variables for the population-level response variables. The coefficient of variation was considered for these variables because seasonal climate variability may be important for explaining amphibian population trends [44, 45]. We also calculated winter snowpack as another potential indicator of winter conditions. Minimum daily temperatures were selected as the temperature metric because 1) minimum temperatures (i.e., nightly temperatures) are likely to be more important to the physiology and movements of nocturnal, fossorial amphibians like spotted salamanders [26, 27] and 2) preliminary analyses revealed that maximum temperatures had considerably lower performance for all analyses compared with minimum temperatures.

A total of 12 climate variables were considered for analysis, which were 1) migration period mean minimum daily temperature (°C; SM_temp), 2) migration period minimum daily temperature seasonality (CV; SM_temp_CV), 3) migration period mean precipitation (mm/day; SM_pre), 4) migration period precipitation seasonality (SM_pre_CV), 5) summer mean minimum daily temperature (SU_temp), 6) summer minimum daily temperature seasonality (SU_temp_CV), 7) summer mean precipitation (SU_pre), 8) summer precipitation seasonality (SU_pre_CV), 9) winter mean minimum daily temperature (WI_temp), 10) winter minimum daily temperature seasonality (WI_temp_CV), 11) winter mean snowpack (WI_snow) and 12) winter snowpack seasonality (WI_snow_CV).

### Data analyses

Prior to performing analyses, we excluded one year of data (2012) from the 23-year dataset due to insufficient data collection. Hence, results documented hereafter for population size, sex ratios, and migration timing includes 22 years of monitoring. Four other years were excluded for the body size analyses (1997–2000) because measurements were incomplete during those years. We evaluated the impacts of seasonal climatic conditions on three elements of spotted salamander population characteristics and two migration timing statistics. Population variables included 1) the total number of migrating salamanders, 2) the snout-vent length (SVL) of both male and female salamanders, and 3) male to female sex ratios. The migration timing statistics included 1) the 95^th^ percentile migration start date for male and female salamanders and 2) the length of the observed migration window for males and females based on the respective starting and ending migration date for each sex. The starting and ending dates for male and female salamanders was based on the 95^th^ percentile threshold defined above for the Spring migration period.

We used autoregressive moving average (ARMA) models to evaluate whether the salamander population characteristics (abundance, sex ratios, body size, migration timing) and seasonal climate variables have changed across time. ARMA models compute a moving average that can estimate trends for datasets even with years of missing data. Using year as a lone independent variable, we added an ARMA correlation structure to general least squares regressions (GLS function; R version 3.2.3; [46]) for all climate variables and population characteristics in order to account for the temporal autocorrelation commonly observed in time series data [33, 47]. The parameters for each respective ARMA model (*p* and *q*) were determined using the autocorrelation and partial autocorrelation functions (ACF and PACF functions; R v. 3.2.3).

We then used a series of generalized linear models (GLM function; R v. 3.2.3) and an information-theoretic framework [48, 49] to evaluate the relationships between the population characteristics of migrating adult salamanders and seasonal climate conditions. We established a set of candidate models *a priori* in order to identify the most parsimonious set of climatic predictors for each response variable. Candidate models for all response variables were composed of 1) simple linear models with single climatic variables, 2) multiple regression models testing for two-way interactive effects for all variable combinations within each of the three ‘seasonal periods’ of interest (summer conditions; winter conditions; migration period conditions), 3) three multiple regression models testing for additive effects of all climatic variables within each of the three seasonal periods, and 4) a global model with additive effects of all seasonal variables combined.

We also tested for time-lag effects in abundance, body size, and sex ratios to better evaluate the potential for delayed changes yet to be observed in population characteristics, since climatic effects may not be immediate [50]. We thus performed three separate information-theoretic analyses for these population characteristics: one for seasonal climatic conditions prior to spring migrations (*t*), one for seasonal conditions two years prior to annual migrations (*t*– 2), and one for seasonal conditions three years prior (*t*– 3). Models were only compared for the same time-lag and not across time-lags because sample sizes differed for each approach (i.e., data on springtime migration conditions were not available for the years prior to beginning our study). Two- and three-year time-lags were tested because this is the time to sexual maturity for spotted salamanders [37]. Finally, we also included abundance as a covariate in models testing for body size effects, since density-dependent effects may be more important than climate for explaining body size changes [51]. All candidate models considered are listed in S1 and S2 Tables. All of the climate variables were treated as fixed effects and none were highly correlated with each other (all Pearson’s |*r*| ≤ 0.54).

Differences in the nature of each response variable required specifying different error distributions across the generalized linear models [47]. We used a negative binomial model (glm.nb function; R v.3.2.3) for discrete count data because annual abundance was highly over-dispersed (i.e., variance was very large relative to the mean). Similarly, the discrete data associated with migration date (day of year) and migration window length (number of days) were modeled with a Poisson distribution and a log-link function. Sex ratios and body sizes were analyzed with a Gaussian (normal) distribution. Model residuals and response variables were tested for normality using a Shapiro-Wilks test and diagnostic plots were visually evaluated to ensure the assumptions of homoscedasticity and normality for linear models were not violated. The CV of winter temperatures and the CV of migration period temperatures were log-transformed to meet these assumptions. Residual variation in models did not display any patterns of temporal autocorrelation, and thus we did not need to account for autocorrelation within the structure of these models.

Candidate models were evaluated using Akaike information criterion corrected for small sample sizes (AIC_c_) because the response variable to predictor ratio was small [47]. Best candidate models for each time-lag analysis were ranked according to ΔAIC_c_ (low values being better fit) and Akaike weights (*w*_i_). We only considered models with *w*_i_ ≥ 0.1 for interpretation. We calculated an evidence ratio (ER; [49]) by dividing the top ranked models *w*_i_ by the second ranked models *w*_i_ for each time-lag. The ER provides an estimate for the likelihood of the hypotheses associated with the top ranked model over the hypotheses of the second ranked mode, with higher values indicating a greater likelihood of support for the first model over the second. We also calculated pseudo-*R*-squared values for the best candidate Poisson and negative binomial models to quantify the amount of variation in salamander population characteristics explained by climatic variables. The significance level of all tests was set to α < 0.05.

## Results

**Fig 1:**
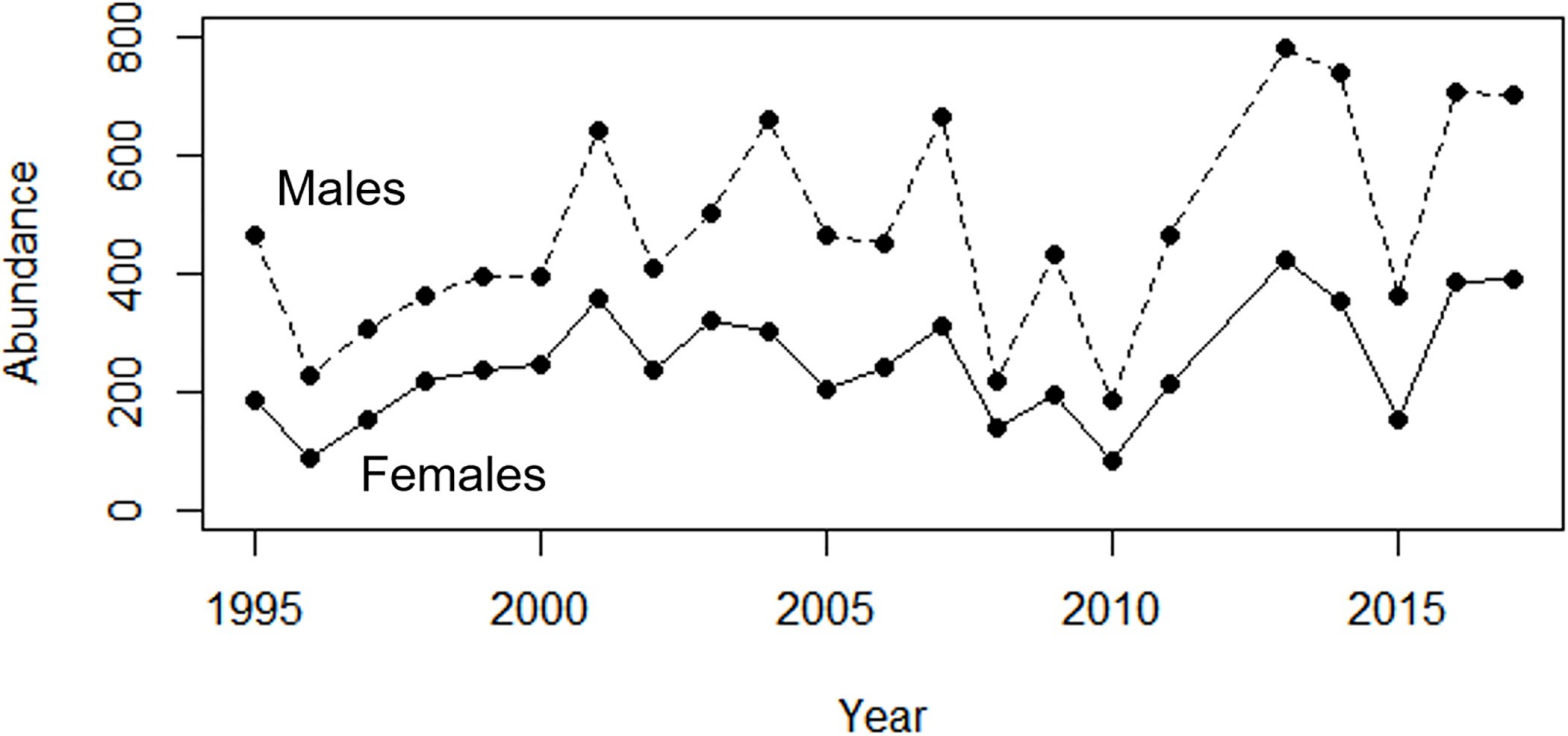
Number of migrating adult salamanders across the 23-year study period. Trends are shown for both males (dashed line) and females (solid line).

**Fig 2:**
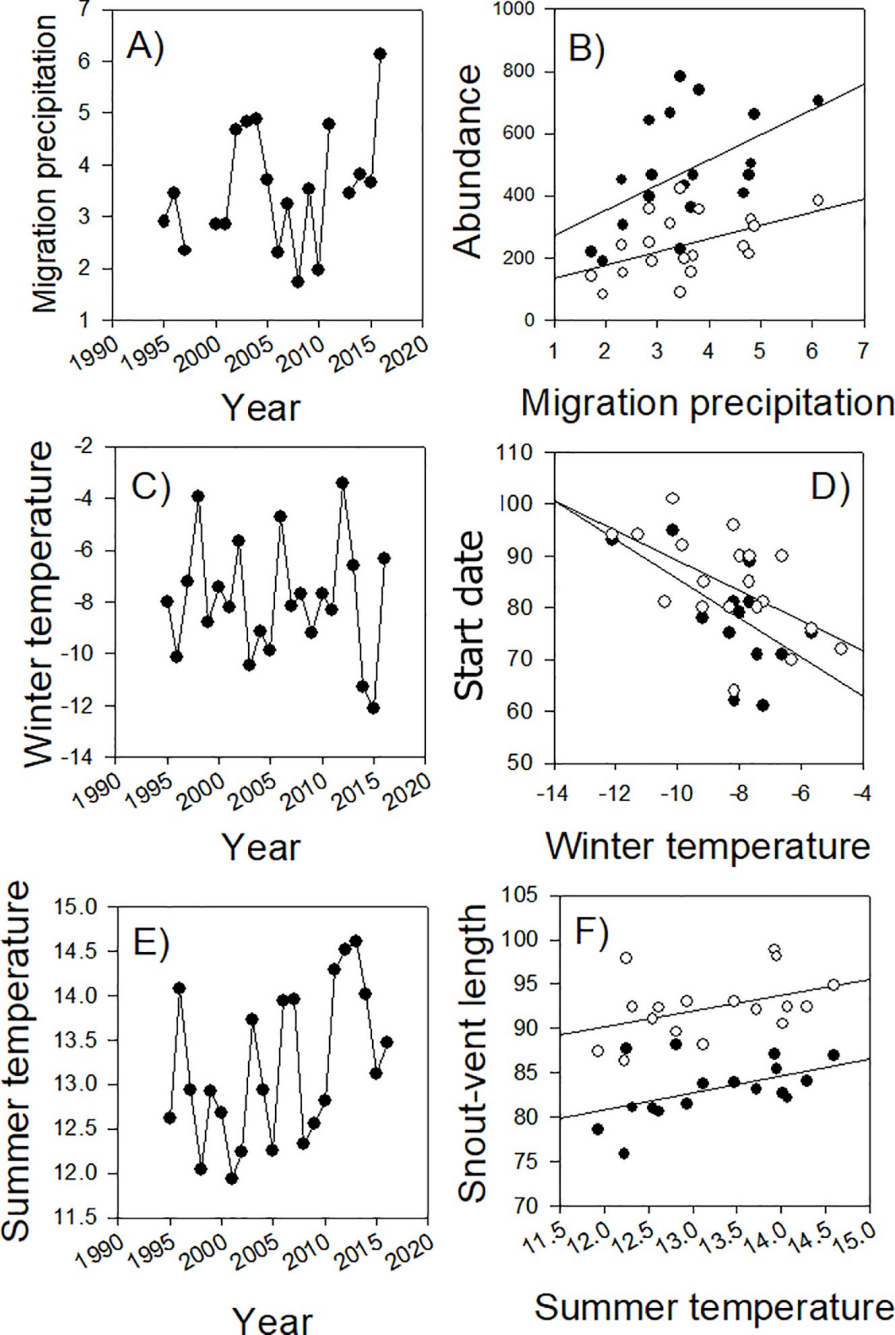
Trends and relationships between seasonal climatic variables and salamander population characteristics. Annual variation in winter-spring transition precipitation (A), minimum winter temperatures (C), and minimum summer temperatures (E). Variation in the total numbers of male (closed circles) and female (open circle) salamander migrants as a function of migration period precipitation (B). The migration start date for male (closed circles) and female (open circles) salamanders based on the 95^th^ percentile arrival day of year (DOY) as a function of minimum previous winter temperature (D). Male (closed circles) and female (open circles) snout-vent lengths (SVL) as a function of minimum previous summer temperatures (F).

There were no long-term shifts in sex ratios, body size or migration timing of spotted salamanders across the 23-year study based on the ARMA models (all *P* ≥ 0.105). There was a weak trend of increasing abundance in male salamanders across the study period (value = increase of 11.6 males per year, *t* = 2.15, SE = 5.42, *P* = 0.044), but no trend for female salamanders (value = 5.7, *t* = 1.64, SE = 3.5, *P* = 0.116; Fig 1). Despite increasing summer minimum temperatures across the study period (value = 1.0°C increase per decade; *t* = 2.45, SE = 0.03, *P* = 0.023; Fig 2E), there was no increase in winter temperatures (value = -0.1, *t* = -0.39, SE = 0.19, *P* = 0.699; Fig 2C). The only other climatic change observed through time was decreasing variability in summer precipitation (value = -0.02, *t* = -3.69, SE = 0.01, *P* < 0.001). To better evaluate changes during the winter-spring transition period when the salamanders migrate, we evaluated trends in minimum temperature and precipitation during 30 days prior and 30 days after the median arrival date of salamanders for each year. There was again no increase in minimum temperatures (value = 0.06, *t* = 1.55, SE = 0.04, *P* = 0.136) or a change in precipitation (value = 0.01, *t* = 0.14, SE = 0.03, *P* = 0.894; Fig 2A) during the winter-spring transition when migration occurs.

The annual abundance of migrating adults was most consistently explained by the amount of precipitation during the migration period (Table 1). The top model for abundance with no time-lag, which was 3.2 times better supported than the second ranked model, included only migration period precipitation, with greater precipitation associated with more adult migrants (pseudo-*R^2^* = 0.24, *P* = 0.004; Fig 2B). Partitioning the abundance into the two sexes revealed the same pattern, with the top model for the number of migrating males (pseudo-*R^2^* = 0.24) and females (pseudo-*R^2^* = 0.22) best explained by the amount of precipitation during the migration period. Furthermore, there was a strong relationship between migration period precipitation and migrant abundance for the two-year time-lag (pseudo-*R^2^* = 0.50, *P* < 0.001; S2 Fig), which was 45 times better supported than the second ranked model. There was also a negative relationship between variability in summer precipitation and migrant abundance for the three-year time-lag (pseudo-*R*^2^ = 0.21, *P* = 0.021) and a weak positive effect of minimum previous summer temperatures on migrant abundance (pseudo-*R*^2^ = 0.15, *P* = 0.055), suggesting that these long-term climatic trends may be associated with the long-term trend of increasing male abundance.

**Table 1:** Top ranked generalized linear regression models for total abundance (for Akaike model weights [*w*_*i*_] > 0.1) based on no time-lag (*t*), a two-year time-lag (*t*– 2), and a three-year time-lag (*t*—3). Models were ranked based on differences in AIC corrected for small sample size (ΔAIC_c_), weights and evidence ratios (*ER*). Climate variable abbreviations can be found in the text or S1 Table.

Time-lag	Model (observed relationships)	Pseudo-*R^2^*	AIC_c_	ΔAIC_c_	*w*_i_	*ER*
*t*	SM_pre (+)**	0.24	308.9	0	0.42	3.23
	SU_temp (+)	0.15	311.3	2.4	0.13	-
*t*– 2	SM_pre (+)***	0.50	258.5	0	0.90	45.00
*t*– 3	SU_pre_CV (-)*	0.21	255.5	0	0.22	1.57
	SU_pre (-), SU_pre_CV (-), SU_pre × SU_pre_CV (+)	0.45	256.3	0.8	0.15	-
	SU_pre (-)*	0.17	257.0	1.5	0.14	-

Climate variable significance is indicated by

*(*P* < 0.05)

** (*P* <0.01), and

*** (*P*<0.001).

Model results for male to female sex ratios were the weakest of all population characteristics (Table 2). Wet summers in the previous year (*R^2^* = 0.22, *P* = 0.028), cold winters in the previous year (*R^2^* = 0.22, *P* = 0.028), and low temperature variation within the migration period were associated with high (male-biased) sex ratios (*R^2^* = 0.18, *P* = 0.045). There were also strong interactive effects for both the no time-lag and three-year time-lag model between summer temperature and summer precipitation conditions, with warm summers driving greater male-biased sex ratios during wet summers (Table 2).

**Table 2:** Top ranked generalized linear regression models for sex ratios (for Akaike model weights [*w*_*i*_] > 0.1) based on no time-lag (*t*), a two-year time-lag (*t*– 2), and a three-year time-lag (*t*—3). Models were ranked based on differences in AIC corrected for small sample size (ΔAIC_c_), weights and evidence ratios (*ER*). Climate variable abbreviations can be found in the text or S1 Table.

Time-lag	Model (observed relationships)	*R^2^*	AIC_c_	ΔAIC_c_	*W*_i_	*ER*
*t*	SU_pre (+)*	0.22	11.3	0	0.17	1.00
	WI_temp (-)*	0.22	11.4	0.0	0.17	-
	SU_pre (+)*, SU_temp (+)*, SU_pre × SU_temp (-)*	0.30	12.0	0.7	0.13	-
	SM_temp_CV (-)*	0.18	12.3	1.0	0.11	-
*t*– 2	Winter_snow (+)	0.15	7.3	0	0.18	1.50
	Winter_snow_CV (+)	0.11	8.1	0.8	0.12	-
*t*– 3	SU_temp (+)**, SU_temp_CV (+)**, SU_temp × SU_temp_CV (-)**	0.33	5.4	0	0.22	1.69
	SU_pre (+)**, SU_pre_CV (+)*, SU_pre × SU_pre_CV (-)*	0.28	6.5	1.1	0.13	-

Climate variable significance is indicated by

*(*P* < 0.05)

** (*P* <0.01), and

*** (*P*<0.001).

**Fig 3:**
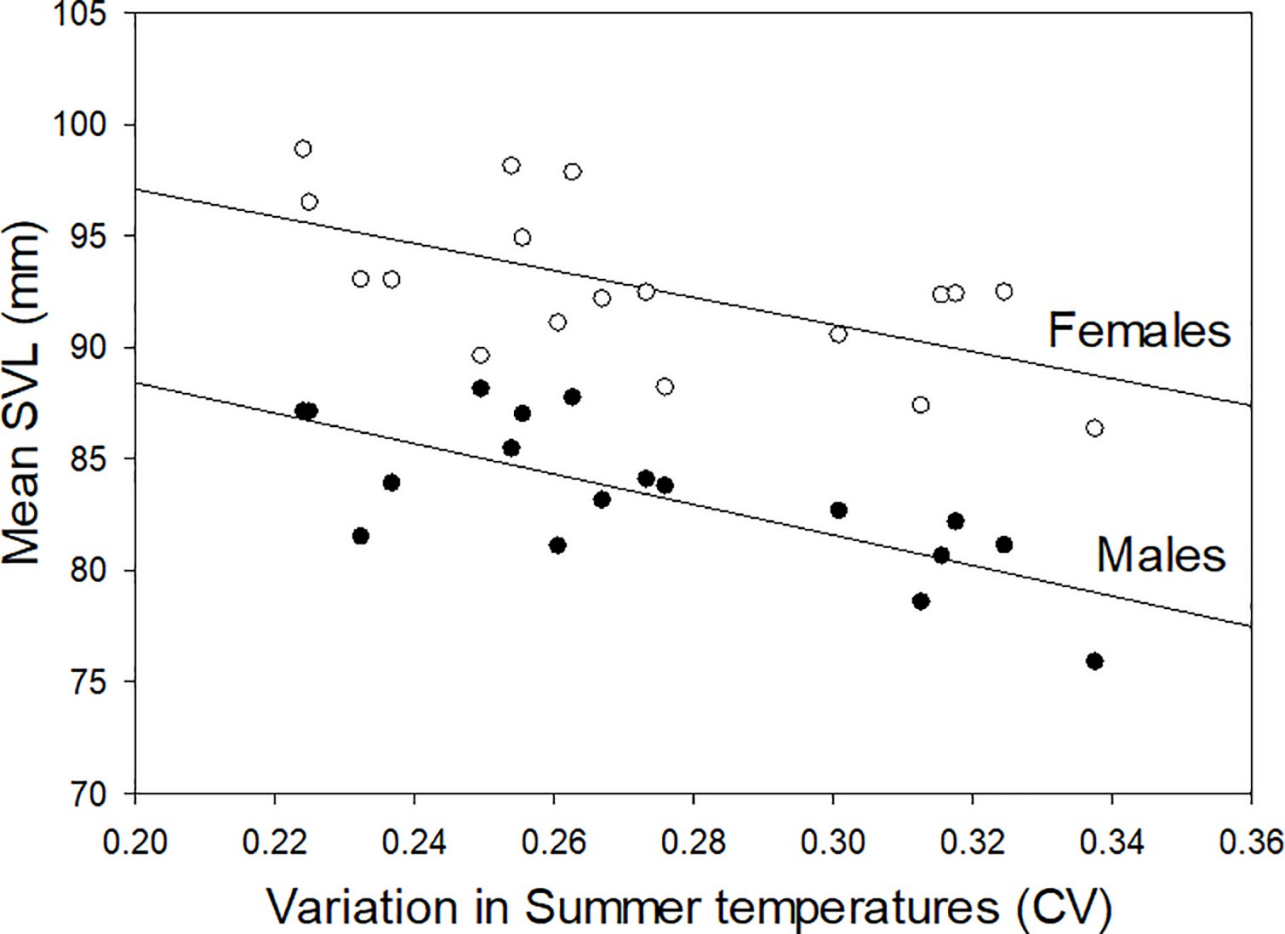
Relationship between the coefficient of variation in minimum previous summer temperatures and the snout-vent length (SVL) of migrating adults. Regression lines are shown for males (closed circles) and females (open circles).

Summer conditions in the previous year explained the highest variation in both male and female body sizes (highest *R^2^*; Tables 3 and 4), with larger migrating adult body sizes following warm summers (Fig 2F). The top ranked model for female body size with no time-lags included all summer climate variables, which had 8.9 times more support than the second ranked model. Notably, the coefficient of variation in summer minimum temperature was a strong predictor of both male and female body sizes, with high variations in summer temperatures correlated with small body sizes of males (*R^2^* = 0.54, *P* < 0.001) and females (*R^2^* = 0.37, *P* = 0.007; Fig 3) in the following year. In contrast, there were no significant effects from the time-lag models or for density-dependent effects from migrant abundance.

**Table 3:** Top ranked generalized linear regression models for male snout-vent length (for Akaike model weights [*w*_*i*_] > 0.1) based on no time-lag (*t*), a two-year time-lag (*t*– 2), and a three-year time-lag (*t*—3). Models were ranked based on differences in AIC corrected for small sample size (ΔAIC_c_), weights and evidence ratios (*ER*). Climate variable abbreviations can be found in the text or S2 Table.

Time-lag	Model (observed relationships)	*R^2^*	AIC_c_	ΔAIC_c_	*W*_i_	*ER*
*t*	SU_temp_CV (-)***	0.54	87.2	0	0.71	10.10
*t*– 2	WI_temp (-)	0.20	84.5	0	0.21	1.20
	WI_temp_CV (-)	0.12	86.0	1.5	0.10	-
*t*– 3	SM_temp (-)	0.15	85.5	0	0.13	1.00
	WI_snow_CV (-)	0.15	85.5	0	0.13	-

Climate variable significance is indicated by

*(*P* < 0.05)

** (*P* <0.01), and

*** (*P*<0.001).

**Table 4:** Top ranked generalized linear regression models for female snout-vent length (for Akaike model weights [*w*_*i*_] > 0.1) based on no time-lag (*t*), a two-year time-lag (*t*– 2), and a three-year time-lag (*t*– 3). Models were ranked based on differences in AIC corrected for small sample size (ΔAIC_c_), weights and evidence ratios (*ER*). Climate variable abbreviations can be found in the text or S2 Table.

Time-lag	Model (observed relationships)	*R^2^*	AIC_c_	ΔAIC_c_	*W*_i_	*ER*
*t*	SU_temp (+)*, SU_temp_CV (-)*, SU_pre (+)*, SU_pre_CV (+)**	0.66	91.1	0	0.61	8.90
*t*– 2	WI_temp (-)	0.23	86.8	0	0.20	1.05
	SU_pre (+)	0.22	86.9	0.1	0.19	-
*t*– 3	WI_temp (-)	0.14	88.5	0	0.13	1.08
	WI_snow_CV (-)	0.12	88.7	0.2	0.12	-
	Abundance (+)	0.11	88.9	0.4	0.11	-
	WI_temp_CV (-)	0.10	89.0	0.5	0.10	-

Climate variable significance is indicated by

*(*P* < 0.05)

** (*P* <0.01), and

*** (*P*<0.001).

Although we did not observe a trend towards earlier migration dates across the 23 years in the ARMA models, winter temperatures had a significant effect on migration timing (Table 5). Both males (pseudo-*R^2^* = 0.59, *P* < 0.001) and females (pseudo-*R^2^* = 0.38, *P* = 0.002) were more likely to migrate early following warm winters (Fig 2D). Coefficient of variation in minimum winter temperatures also had a significant effect on migration timing, with high variability (i.e., uncertainty) in winter temperatures associated with early migrations for females (pseudo-*R^2^* = 0.47, *P* < 0.001) and males (pseudo-*R^2^* = 0.53, *P* < 0.001). The coefficient of variation in precipitation during the migration period was the most consistent predictor of migration window length, and was positively associated with the breeding window length of both male (pseudo-*R^2^* = 0.42, *P* < 0.001) and female salamanders (pseudo-*R^2^* = 0.37, *P* < 0.001). Hence, large variation in precipitation associated with more periods of no rainfall resulted in more migration delays.

**Table 5:** Top ranked generalized linear regression models for migration timing statistics (for Akaike model weights [*w*_*i*_] > 0.1). Models were ranked based on differences in AIC corrected for small sample size (ΔAIC_c_), weights and evidence ratios (*ER*). Climate variable abbreviations can be found in the text or S1 Table.

Attribute	Model (observed relationships)	Pseudo-*R^2^*	AIC_c_	ΔAIC_c_	*W*_i_	*ER*
Male timing	WI_temp (-)***	0.59	155.6	0	0.55	2.62
	WI_temp_CV (-)***	0.53	157.6	2.0	0.21	-
Female timing	WI_temp_CV (-)***	0.47	155.6	0	0.51	3.00
	WI_temp (-)**	0.38	157.9	2.3	0.17	-
Male window	SM_temp (-)**, SM_pre_CV (+)***, SM_temp × SM_pre_CV (+)*	0.54	163.4	0	0.71	5.46
	SM_temp (-)**, SM_pre (-), SM_temp_CV (+), SM_pre_CV (+)*	0.54	166.9	3.5	0.13	-
	SM_temp_CV (+)**, SM_pre_CV(+)***, SM_temp_CV × SM_pre_CV (-)**	0.50	167.0	3.6	0.12	-
Female window	SM_pre_CV (+)***	0.37	168.8	0	0.49	2.23
	SM_temp (-), SM_pre_CV (+)***, SM_temp × SM_pre_CV (+)	0.41	170.4	1.6	0.22	-
	SM_temp_CV (+)*, SM_pre_CV(+)***, SM_temp_CV × SM_pre_CV (-)*	0.41	170.5	1.7	0.20	-

Climate variable significance is indicated by

*(*P* < 0.05)

** (*P* <0.01), and

*** (*P*<0.001).

## Discussion

Three frequently documented ecological responses of ectothermic organisms to climate change are: 1) range shifts, 2) changes in phenology (i.e., migration timing), and 3) changes in body size [10, 11, 52]. As in previous studies, we observed year-to-year variation in the migration timing and body size of spotted salamanders that was coincident with inter-annual variation in temperature and precipitation [7, 9, 27, 28]. However, we did not observe long-term, climate-induced shifts in population characteristics or migration timing of this species across the 23-year study. The absence of climate-induced population changes reflects a lack of change in the seasonal climate conditions important for explaining this species population characteristics. Our results provide insight into why different studies reach different conclusions about amphibian responses to climate change [31, 32]. Specifically, species vulnerability to climate change will depend on how different climate variables are changing at different rates across regions [53].

A striking difference between our results and those of other studies with ectothermic taxa is the absence of a multi-decadal trend towards advanced migration timing [27, 28, 39, 40]. We observed early salamander migrations in warm winters, which is consistent with the prediction that long-term warming would lead to earlier migration. However, we did not observe a long-term warming trend during winter at our study site (Fig 2C), despite observing clear warming trends during the summer and autumn seasons (latter defined as September 22–December 21; value = 0.7°C increase per decade; *t* = 10.53, SE = 0.01, *P* < 0.001). Climate change models in our study region of northwestern Pennsylvania predict future winter warming [34], which could lead to an eventual advancement in migration timing for this species.

The consequences of altered migration timing include a decoupling of community dynamics [54] and asynchrony in the arrival time of sexes [26]. It is well documented that male ambystomatid salamanders arrive earlier than females to breeding ponds [37], and that males and females have different temperature thresholds for initiating migration [25]. Given that higher variability in migratory precipitation and temperature cues resulted in longer breeding windows (i.e., migratory delays; Table 5), extended dry periods in the early versus late migration window will have disproportional effects on each sex. Extended drying during migrations may be most likely to affect the migration of females, which frequently skip breeding during years of sub-par migration conditions (i.e., low precipitation; [23, 38, 55]). However, in our study, the pattern of early arrival during warm winters was very similar for males and females (Fig 2D), suggesting that climate warming is unlikely to lead to further asynchronous arrival between the two sexes.

Similar patterns were observed for how inter-annual variation in climate influence body size, despite the absence of an overall body size shift during the study period (Fig 2E and 2F). Although recent evidence suggests that climate change can lead to body size reductions in amphibians [7, 9], others have offered alternative explanations [56] or have found increases in body size [10, 57]. We did observe a positive relationship between body size and summer temperatures, which is contrary to our predictions and those of other studies. Caruso et al. [9] used biophysical modeling to show that salamander metabolic expenditure has increased through time due to rising temperatures, and thermal stress is a likely physiological mechanism for body size reductions. However, maximum summer temperatures across all study years (range: 24.2–28.1°C) never approached the reported thermal critical maxima for spotted salamanders and were thus probably rarely thermally stressed (~35°C [58]).

The absence of long-term shifts in body size could be due to several alternative hypotheses. First, climate-induced changes of body size may be delayed by several years, especially when considering that size at metamorphosis is correlated to size at maturity [59, 60]. Second, amphibian body sizes have also been shown to be density-dependent, suggesting that body sizes may be driven by relationships between resource availability and abundance [51]. However, we found no significant time-lag effects and no relationships with the abundance of returning salamanders to support these hypotheses (Tables 3 and 4). Third, we might not be adequately capturing demographic changes in the population that could mask climate-induced body size changes. The long-term increases in abundance may be a result of increasing survival and high breeding probabilities for young, small individuals that could also be affecting body size patterns [23, 37]. The fourth hypothesis, which we provide support for below, is that another important climatic factor has additionally strong effects that is preventing a body size shift despite a prominent warming trend (Fig 2E).

The strongest response we observed was a significant negative relationship between salamander body size and within-year fluctuations in summer temperatures (Fig 3), a climate variable that has not experienced a change through time (value = -0.02, *t* = 0.38, SE = 0.01, *P* = 0.712). Although the mechanisms underlying this relationship remain unknown, one hypothesis is that the energetic costs associated with maintaining optimal metabolic temperatures should be higher under fluctuating rather than constant external temperatures. Amphibians have low resting metabolic rates, which make them well adapted for storing energetic reserves [20]. However, alternating between high and low metabolic states or constantly acclimating across temperatures could require significant energetic expenditure and can have profound physiological consequences during both active and hibernation periods [12, 22, 61]. Identifying the mechanisms of how climate change and temperature variability within seasons impacts the physiology and body size of amphibians is likely to be important for understanding how climate variability affects amphibians (e.g., seasonal extremes; [44]).

An additional consequence of climate change to amphibians could be local population declines or extirpations [14, 15, 16]. Perhaps not surprisingly, the dominant predictor for the number of returning spotted salamanders was precipitation during the migration period, which has long been recognized as the primary stimulant for initiating migrations in ambystomatid salamanders [24, 25, 37]. Reduced springtime precipitation from climate change would likely reduce the number of migrating adults due to insufficient migratory cues and conditions, whereas reduced precipitation during the dry season (i.e., summer) could affect population size through larval desiccation if ponds dry early [22, 23]. In fact, the influence of migration period precipitation in the two-year time-lag analyses suggests an important link between migrant abundance and recruitment that affects future abundance (S2 Fig). Once again though, we observed no trends related to precipitation that led to obvious long-term changes in population size (Fig 2A and 2B). Finally, climate warming can also have positive effects on population size [29], and we observed a potential link between summer warming and increased male abundance across our study period (Table 1). Climate change models in our study region predict increased precipitation for all seasons [34], and while this could have an overall benefit to the species by further increasing population sizes, it may also contribute to a larger skew in sex ratios.

Explanatory power was weakest for models evaluating relationships between climate and the sex ratio of male to female migrants, in part because male and female abundances varied similarly with precipitation during the spring migration. Sex ratios of migrants in a given year are normally male-biased and represent an important proxy for the ‘skipping breeding’ behavior observed in females [38]. There was substantial inter-annual variation in sex ratios (range: 1.56–2.59) resulting in fewer females (higher ratios) migrating during colder winters and wetter summers; the latter of which was observed in both the no time-lag and three-year time-lag analyses (Table 2). However, these responses appear contradictory; fewer females migrated during apparently unfavorable conditions in one instance (colder winters) and under apparently favorable conditions in another (wetter summers; [31]). Given the weak explanatory power of these models, our results do not provide strong insight into how long-term climate change may affect variation in sex ratios.

The responsiveness of different amphibian species or populations to a changing climate will depend on how a variety of species-specific characteristics determine individual sensitivity and adaptability to climate change. Certain populations may be at higher risk depending upon their geographical position within the species distribution (i.e., a climatic extreme; [62]), as well as their total range sizes. Spotted salamanders have a large range size across most of eastern North America and this population does not lie at a distributional limit; both of which may make them less susceptible to climatic changes than species with smaller range sizes [63]. With respect to phenology, changes in migration timing will depend on seasonal differences in climate change, such as whether climate warming occurs for autumn versus spring migrants (e.g., [27]). From an eco-physiological perspective, pond-breeding ambystomatids might be less susceptible to climate change than stream-dwelling, plethodontid salamanders because the latter are lungless and particularly sensitive to changes in moisture and temperature. Species and populations at higher elevations are also more susceptible to range contractions and extirpations than those at lower elevations [64]. Indeed, documented climate change impacts on high elevation plethodontids have been more dramatic than that of ambystomatids [9, 14, 17, 32]. Different species will also vary in their susceptibility to indirect effects of climate change from other stressors, such as susceptibility to infectious diseases [5, 12, 16, 45]. Even within the same habitats, some species will be more vulnerable to this interaction whereas others serve mainly as vectors and are only weakly affected [29].

Recent meta-analyses concluded that amphibian responses to climate change will be idiosyncratic and context-dependent [31, 32]. Because species and populations are likely to differ in responses to climate change based on differences in life-history, life stage, and physiology, multi-species assessments [27, 29] and large meta-analyses [31, 32] will be critical in helping ecologists formulate stronger inferences about climate-induced changes in amphibians. Although a caveat of our study is that we only evaluated a single species at a single location, our results are in general agreement with the conclusion of strong idiosyncratic responses in amphibians. Even though spotted salamanders were highly responsive to annual variation in seasonal climate, the absence of consistent long-term changes in important climatic variables appears to have precluded long-term changes in population characteristics and migration timing.

In conclusion, more attention should be given to understanding how seasonal climate change differences can explain the responsiveness of amphibians to climate change. Many studies have identified climate-induced changes in amphibians, which reflect the sensitivity of this taxonomic group to climatic factors [31]. Despite that sensitivity, idiosyncratic responses are prevalent throughout the literature because the responsiveness of a given species to climate change will depend on how the ecology of the species (e.g., phenology, life history) interacts with climatic changes at a given location [53]. Linking fine-scale, localized climatic changes (e.g., seasonal) to species or populations should thus improve our ability to explain why there is so much variation in responses across amphibian taxa [33].

## Supporting information

S1 Fig The five ponds within the Bousson Environmental Research Reserve (BERR) that were annually sampled for spotted salamanders during a 23-year study from 1995–2017. Inset map shows the location of the study site within the state of Pennsylvania (PA) relative to the northeastern United States (e.g., states of New York [NY] and Maine [ME]).
(DOCX)

S2 Fig Relationship between annual spotted salamander abundance and the amount of precipitation during the migration period from two years prior (*t*– 2). (DOCX)

S1 Table Set of 34 candidate models used for the generalized linear regression models of abundance, sex ratio’s, and migration timing for times *t*, *t–* 2, and *t–* 3. CV refers to the coefficient of variation estimated for each climate variable. × indicates models with interactive effects and + indicates models with additive effects.
(DOCX)

S2 Table Set of 50 candidate models used for the generalized linear regression models of body size for times *t*, *t–* 2, and *t*– 3. CV refers to the coefficient of variation estimated for each climate variable. × indicates models with interactive effects and + indicates models with additive effects.
(DOCX)

S1 File Spreadsheet with the annual spotted salamander and climate data from 1995–2017 used in analyses for this paper. (CSV)
