# Supplementary material for: Seasonal differences in climate change explain a lack of multi-decadal shifts in population characteristics of a pond breeding salamander
Source: PLoS One. 2019 Sep 6;14(9):e0222097. doi: 10.1371/journal.pone.0222097 (PMC6730874; doi:10.1371/journal.pone.0222097)

**S1 Fig. The five ponds within the Bousson Environmental Research Reserve (BERR) that were annually sampled for spotted salamanders during a 23-year study from 1995-2017.** Inset map shows the location of the study site within the state of Pennsylvania (PA) relative to the northeastern United States (e.g., states of New York [NY] and Maine [ME]).


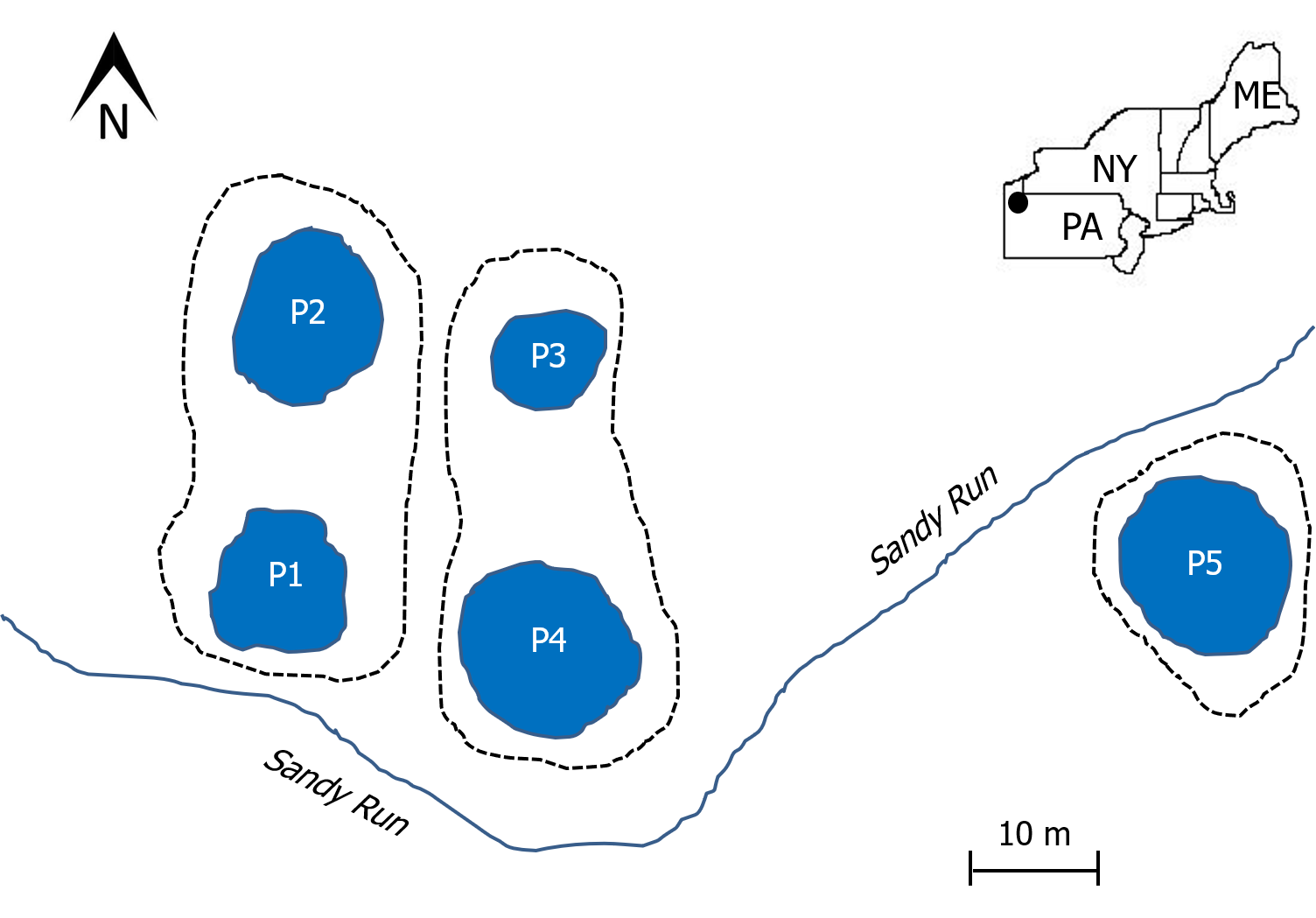

Supplement: S1 Fig — Inset map shows the location of the study site within the state of Pennsylvania (PA) relative to the northeastern United States (e.g., states of New York [NY] and Maine [ME]). (DOCX) [file pone.0222097.s001.docx]
