# Supplementary material for: Seasonal differences in climate change explain a lack of multi-decadal shifts in population characteristics of a pond breeding salamander
Source: PLoS One. 2019 Sep 6;14(9):e0222097. doi: 10.1371/journal.pone.0222097 (PMC6730874; doi:10.1371/journal.pone.0222097)
Supplement: S2 Fig — (DOCX) [file pone.0222097.s002.docx]

**S2 Fig. Relationship between annual spotted salamander abundance and the amount of precipitation during the migration period from two years prior (*t* – 2).**
