# Supplementary material for: Seasonal differences in climate change explain a lack of multi-decadal shifts in population characteristics of a pond breeding salamander
Source: PLoS One. 2019 Sep 6;14(9):e0222097. doi: 10.1371/journal.pone.0222097 (PMC6730874; doi:10.1371/journal.pone.0222097)
Supplement: S1 Table — CV refers to the coefficient of variation estimated for each climate variable. × indicates models with interactive effects and + indicates models with additive effects. (DOCX) [file pone.0222097.s003.docx]

**S1 Table. Set of 34 candidate models used for the generalized linear regression models of abundance, sex ratio’s, and migration timing for times *t*, *t* – 2, and *t* – 3.** CV refers to the coefficient of variation estimated for each climate variable. × indicates models with interactive effects and + indicates models with additive effects.

| Model Name | *K* (parameters + intercept) |
| --- | --- |
| Summer mean minimum daily temperature (SU_temp) | 2 |
| Summer minimum daily temperature seasonality (SU_temp_CV) | 2 |
| Summer mean precipitation (SU_pre) | 2 |
| Summer precipitation seasonality (SU_pre_CV) | 2 |
| Winter mean minimum daily temperature (WI_temp) | 2 |
| Winter minimum daily temperature seasonality (WI_temp_CV) | 2 |
| Winter mean snowpack (WI_snow) | 2 |
| Winter snowpack seasonality (WI_snow_seasonality) | 2 |
| Migration period mean minimum daily temperature (SM_temp) | 2 |
| Migration period minimum daily temperature seasonality (SM_temp_CV) | 2 |
| Migration period mean precipitation (SM_pre) | 2 |
| Migration period precipitation seasonality (SM_pre_CV) | 2 |
| SU_temp × SU_temp_CV | 4 |
| SU_pre × SU_pre_CV | 4 |
| SU_temp × SU_pre | 4 |
| SU_temp_CV × SU_pre_CV | 4 |
| SU_temp × SU_pre_CV | 4 |
| SU_pre × SU_temp_CV | 4 |
| WI_temp × WI_temp_CV | 4 |
| WI_snow × WI_snow_CV | 4 |
| WI_temp × WI_snow | 4 |
| WI_temp_CV × WI_snow_CV | 4 |
| WI_temp × WI_snow_CV | 4 |
| WI_snow × WI_temp_CV | 4 |
| SM_temp × SM_temp_CV | 4 |
| SM_pre × SM_pre_CV | 4 |
| SM_temp× SM_pre | 4 |
| SM_temp_CV × SM_pre_CV | 4 |
| SM_temp × SM_pre_CV | 4 |
| SM_pre × SM_temp_CV | 4 |
| SU_temp + SU_temp_CV + SU_pre + SU_pre_CV | 5 |
| WI_temp + WI_temp_CV + WI_pre + WI_pre_CV | 5 |
| SM_temp + SM_temp_CV + SM_pre + SM_pre_CV | 5 |
| Global additive effects model | 13 |
